# The effect of ultrasound-guided lung recruitment maneuvers on postoperative atelectasis: a systematic review and meta-analysis of randomized controlled trials

**DOI:** 10.3389/fcvm.2026.1890372

**Published:** 2026-08-12

**Authors:** Yilu Bao, Honghang Lin, Bo Tang, Kai Zhang, Biyun Chen

**Affiliations:** 1 Department of Ultrasound Medicine, The First People’s Hospital of Taizhou, Taizhou, China; 2 Department of Emergency Medicine, The First People’s Hospital of Taizhou, Taizhou, China; 3 Department of Critical Care Medicine, Second Affiliated Hospital of Zhejiang University School of Medicine, Hangzhou, China

**Keywords:** atelectasis, lung recruitment maneuvers, meta-analysis, perioperative, ultrasound-guided

## Abstract

**Background:**

Postoperative atelectasis is common after general anesthesia. Lung ultrasound may help individualize recruitment maneuvers, but the certainty and generalizability of the evidence remain uncertain.

**Methods:**

We searched PubMed, Embase, Scopus, and the Cochrane Library through March 25, 2026 for randomized controlled trials (RCTs) of intraoperative ultrasound-guided lung recruitment maneuvers in adults undergoing elective surgery. The primary outcome was postoperative atelectasis assessed by lung ultrasound. Secondary outcomes were postoperative pulmonary complications (PPCs) and length of hospital stay. A random-effects model was used to calculate pooled risk ratios (RR) and mean differences (MD) with 95% confidence intervals (CIs). The certainty of evidence was assessed using the Grading of Recommendations Assessment, Development and Evaluation approach.

**Results:**

Ten RCTs involving 1,076 patients were included. Ultrasound-guided lung recruitment maneuvers were associated with a lower incidence of postoperative atelectasis (RR, 0.64, 95% CI, 0.52 to 0.77, *P* < 0.0001, I2 = 50.8%). The effect appeared clearer in abdominal surgery than in thoracic surgery. PPCs were not significantly reduced in the primary analysis (RR, 0.70, 95% CI, 0.48 to 1.01, *P* = 0.0571, I2 = 59.2%), and length of hospital stay was statistically but modestly shorter (MD −0.56 days, 95% CI −0.94 to −0.18, *P* = 0.0039, I2 = 67.7%).

**Conclusion:**

Ultrasound-guided lung recruitment maneuvers may reduce postoperative atelectasis, especially in abdominal surgery, but the certainty of evidence is limited by the small evidence base, residual clinical and statistical heterogeneity, operator dependency of lung ultrasound, and inconsistent outcome definitions. The modest reduction in hospital stay should be interpreted cautiously. Larger standardized trials are needed before broad clinical implementation can be recommended.

## Introduction

Postoperative pulmonary complications (PPCs), particularly atelectasis, remain common after general anesthesia and may impair gas exchange, delay recovery, and prolong hospitalization (1–4). Atelectasis is promoted by anesthesia-induced reductions in functional residual capacity, supine positioning, and pneumoperitoneum during laparoscopic surgery (5). Recruitment maneuvers aim to reopen collapsed alveoli by transiently increasing transpulmonary pressure (6). However, conventional protocols often apply fixed pressures without real-time information on lung aeration, creating the risk of either insufficient recruitment or excessive pressure with overdistension, hemodynamic instability, or barotrauma (7, 8).

Point-of-care lung ultrasound offers a bedside method for visualizing perioperative aeration and may allow recruitment pressures to be titrated to individual response (9, 10). Several randomized controlled trials (RCTs) have evaluated ultrasound-guided recruitment maneuvers, but they differ in surgical populations, recruitment protocols, pressure targets, timing, post-recruitment ventilation, and outcome assessment (11–14).

We therefore performed an updated systematic review and meta-analysis to estimate the effect of ultrasound-guided recruitment maneuvers on postoperative atelectasis while explicitly considering the limitations of this heterogeneous evidence base.

## Methods

### Protocol registration and reporting standards

This systematic review and meta-analysis followed the Preferred Reporting Items for Systematic Reviews and Meta-Analyses (PRISMA) 2020 guidelines (Supplementary Material 1) (15). The protocol was registered prospectively on the Open Science Framework before study selection and data extraction (https://osf.io/ebfj3).

### Data sources and search strategy

PubMed, Embase, Scopus, and the Cochrane Library were searched from inception to March 25, 2026 using terms related to ultrasound guidance, lung recruitment, atelectasis, surgery, and randomized trials. The complete search strategy is provided in Supplementary Material 2, and reference lists of eligible studies and relevant reviews were screened manually.

### Study eligibility criteria

Studies were included if they met all of the following criteria:

Population: Adult patients (≥18 years) undergoing general anesthesia for elective surgery.
Intervention: Ultrasound-guided lung recruitment maneuvers performed intraoperatively.
Comparator: Non-ultrasound-guided recruitment maneuvers or standard lung-protective ventilation without active recruitment.
Outcomes: The primary outcome was the incidence of postoperative atelectasis, defined or diagnosed through lung ultrasound assessment. Secondary outcomes included the overall incidence of PPCs and the length of hospital stay.
Study design: RCTs published in English.

Studies involving exclusively pediatric populations, non-randomized trials, case reports, review articles, or studies where atelectasis was not confirmed via lung ultrasound were excluded.

### Study selection and data extraction

Two reviewers (Yilu Bao, Honghang Lin) independently screened titles, abstracts, and full texts, extracted study characteristics and outcome data, and resolved disagreements by discussion or consultation with a third reviewer (Kai Zhang). Extracted variables included country, sample size, surgical type, intervention and comparator protocols, recruitment timing, post-recruitment ventilation strategy, and outcome definitions when reported. When data were incomplete or reported in non-standard formats, required statistics were derived from published information or obtained by contacting corresponding authors where feasible.

### Risk of bias assessment

Risk of bias was assessed independently by two reviewers (Yilu Bao, Honghang Lin) using the Cochrane Risk of Bias 2.0 (RoB 2) instrument (16). Any discrepancies in judgment arising between the two reviewers were reconciled through deliberation until mutual consensus was reached.

### Data synthesis and statistical analysis

Risk ratios (RRs) were pooled for dichotomous outcomes and mean differences (MDs) for length of hospital stay, each with 95% confidence intervals (CIs). Random-effects models were used because clinical heterogeneity was expected. Heterogeneity was assessed with Cochran's Q test and quantified using the I^2^ statistic, where values of 25%, 50%, and 75% were predefined as thresholds for low, moderate, and substantial heterogeneity, respectively (17).

Prespecified subgroup analyses were performed based on surgical type (thoracic surgery vs. abdominal surgery), control group intervention (non-ultrasound-guided lung recruitment maneuvers vs. standard protective mechanical ventilation). Leave-one-out sensitivity analyses were performed when sufficient studies were available. Publication bias was explored using funnel plots and Egger's test (18). Trim-and-fill analysis was used only as an exploratory sensitivity method and was not interpreted as proof of an underlying treatment effect (19). We considered additional analyses according to operator expertise, timing of atelectasis assessment, diagnostic criteria, and lung ultrasound scoring methods; however, these analyses were not performed because reporting was inconsistent, categories were sparse and overlapping, and meta-regression would be unreliable with only ten trials. The certainty of evidence for major outcomes was assessed using the Grading of Recommendations Assessment, Development and Evaluation (GRADE) approach (20). Analyses were performed in R using the “meta” and “robvis” packages (21, 22).

## Results

### Study selection and study characteristics

**Figure 1:**
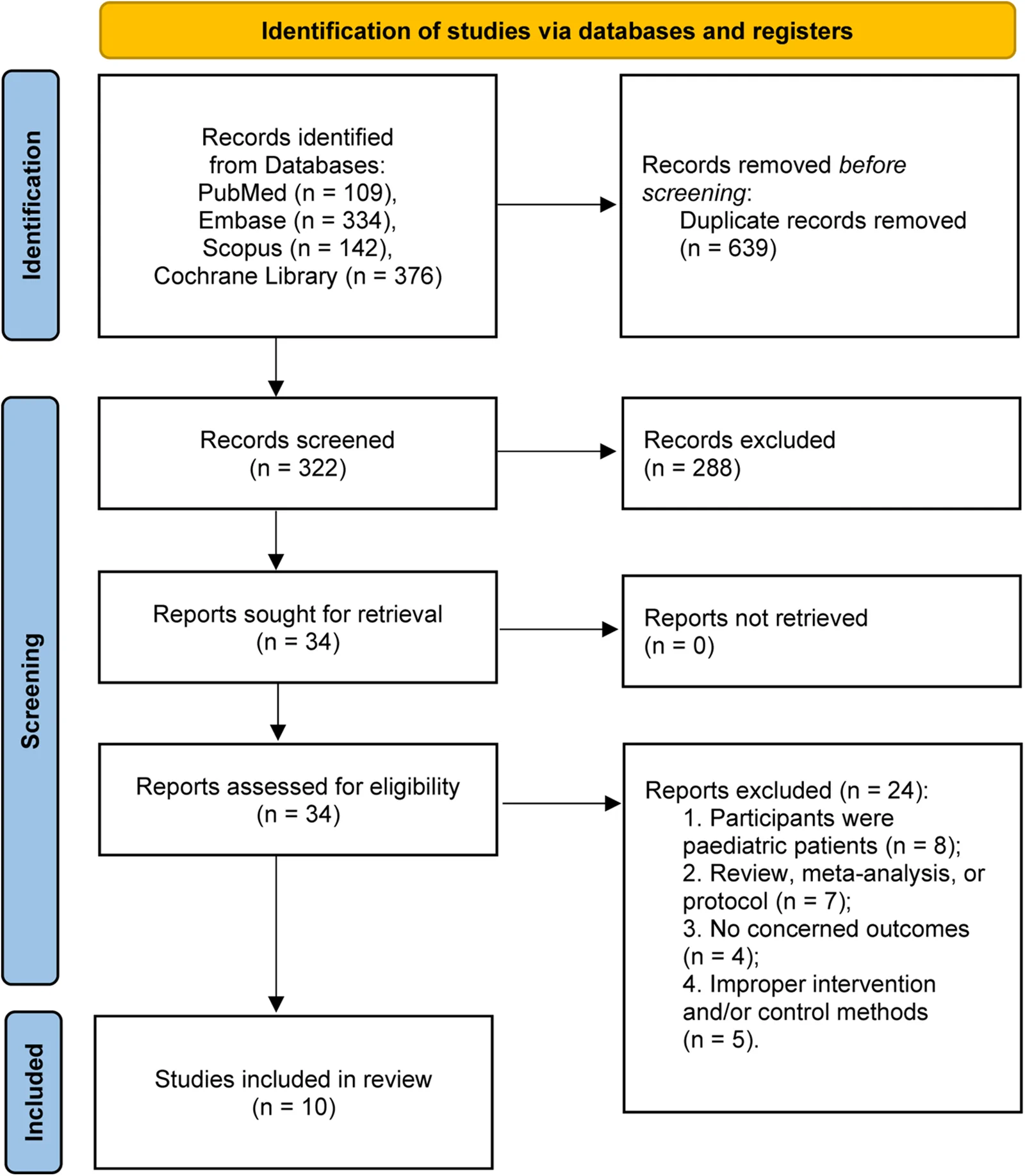
PRISMA 2020 flow diagram for this systematic review and meta-analysis.

The PRISMA flow diagram is shown in Figure 1. Searches identified 961 records; after removal of 639 duplicates, 322 records were screened, 34 full texts were assessed, and 10 RCTs (11–13, 23–29) were included.

The baseline characteristics of the included RCTs are summarized in Table 1. The 10 trials enrolled 1,076 patients (540 intervention, 536 control), with individual sample sizes ranging from 40 to 353 participants. Eight studies were conducted in China (12, 13, 23–25, 27–29) and two in Korea (11, 26). Seven trials enrolled abdominal surgery patients (12, 13, 24–26, 28, 29) and three enrolled thoracic surgery patients (11, 23, 27). Six trials compared ultrasound-guided recruitment with conventional recruitment (11, 12, 23, 26–28), and four compared it with standard protective ventilation without active recruitment (13, 24, 25, 29).

**Table 1:** Characteristics of included studies.

Study, year and country	Sample size	Type of surgery	Intervention	Control	Outcomes
Xu Z et al. 2026, China	77/75	Abdominal surgery	Driving pressure-guided ventilation and lung ultrasound monitoring	Conventional protective ventilation	Atelectasis after extubation, PPCs within 7 days, length of hospital stay
Xu Y et al. 2026, China	179/174	Abdominal surgery	Ultrasound-guided recruitment maneuvers	Conventional recruitment maneuver	Atelectasis with 1 day, PPCs within 7 days, length of hospital stay
Kim BR et al. 2025, Korea	82/84	Thoracic surgery	Ultrasound-guided alveolar recruitment maneuvers	Conventional recruitment maneuver	Atelectasis after extubation, PPCs during hospitalization, length of hospital stay
Wu L et al. 2024, China	30/30	Thoracic surgery	Ultrasound-guided recruitment maneuvers	Conventional recruitment maneuver	Atelectasis after extubation, length of hospital stay
Bai F et al. 2024, China	42/42	Thoracic surgery	Ultrasound-guided recruitment maneuvers	Conventional recruitment maneuver	Atelectasis after extubation, length of hospital stay
Liu T et al. 2023, China	40/40	Abdominal surgery	Ultrasound-guided recruitment maneuvers	Conventional protective ventilation	Atelectasis after extubation, length of hospital stay
Wu X et al. 2022, China	30/30	Abdominal surgery	Ultrasound-guided alveolar recruitment maneuvers	Sustained inflation alveolar recruitment manoeuvres	Atelectasis after extubation, length of hospital stay
Liu Y et al. 2022, China	20/21	Abdominal surgery	Ultrasound-guided stepwise recruitment maneuvers	Conventional protective ventilation	Atelectasis after extubation, length of hospital stay
Yang Y et al. 2021, China	20/20	Abdominal surgery	Ultrasound-guided stepwise recruitment maneuvers	Conventional protective ventilation	Atelectasis after extubation, PPCs during hospitalization, length of hospital stay
Park SK et al. 2021, Korea	20/20	Abdominal surgery	Ultrasound-guided alveolar recruitment maneuvers	Conventional recruitment maneuver	Atelectasis after extubation, length of hospital stay

Important protocol differences were present across trials. Recruitment techniques, pressure targets, timing of maneuvers, lung ultrasound scoring systems, and post-recruitment ventilation strategies varied. Xu Z et al. (13) incorporated individualized positive end-expiratory pressure (PEEP) titration to minimize driving pressure combined with lung ultrasound-guided recruitment, and Liu T et al. (24) evaluated ultrasound-guided recruitment combined with PEEP. Most other studies used fixed PEEP levels or reinstated prior ventilator settings after recruitment.

### Risk of bias assessment

**Figure 2:**
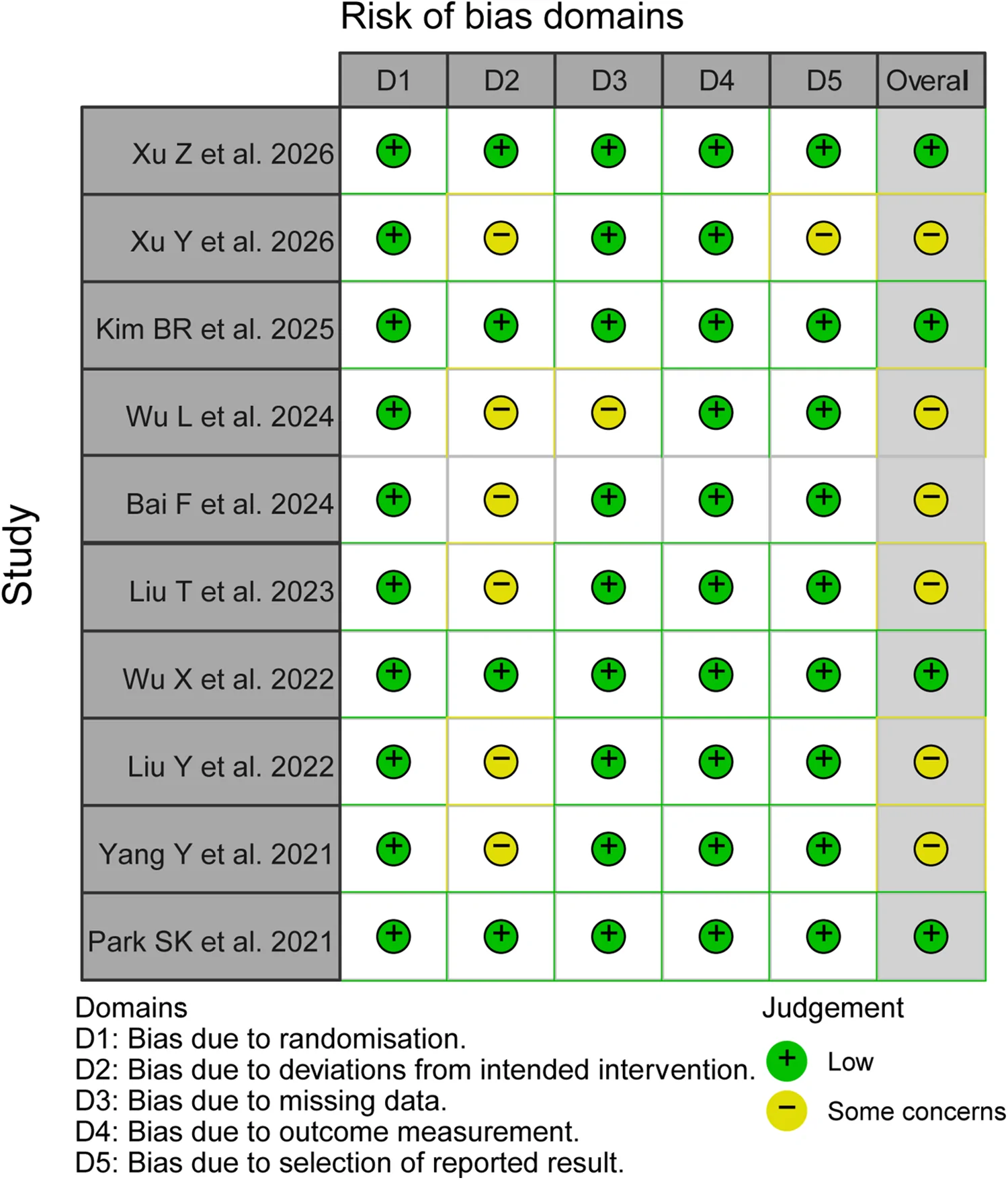
Risk of bias 2 of all included studies.

The methodological quality of the 10 included RCTs was evaluated using the RoB 2 tool (Figure 2). Four trials were judged at low risk of bias and six as having some concerns, mainly because blinding of anesthesiologists was not feasible for intraoperative ventilation interventions. In addition, lung ultrasound acquisition and interpretation are operator-dependent. The included studies did not consistently report operator training, experience, formal competency assessment, or interobserver reliability. This prevented subgroup analysis or meta-regression by operator expertise and limits the interpretation and generalizability of the pooled estimates.

Funnel plot inspection and Egger's test suggested possible small-study effects for atelectasis and PPCs (Supplementary Material 3). Trim-and-fill analyses were treated as exploratory only; they did not remove the underlying concerns about small-study effects, selective reporting, or heterogeneity.

### Primary outcome: postoperative atelectasis

**Figure 3:**
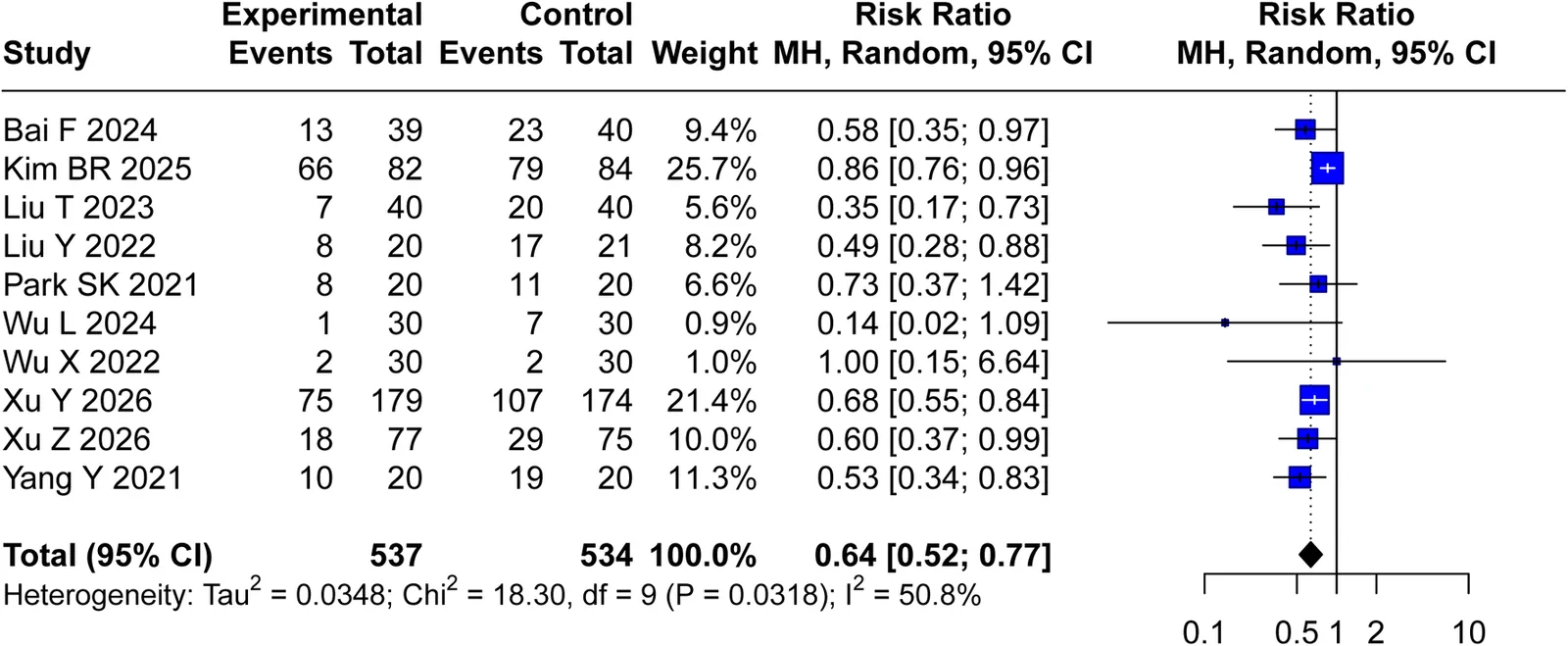
Forest plot comparing the effect of ultrasound-guided lung recruitment maneuvers on incidence of postoperative atelectasis.

The meta-analysis of the 10 included RCTs demonstrated that ultrasound-guided lung recruitment maneuvers were associated with reduced postoperative atelectasis compared with control strategies (RR 0.64, 95% CI 0.52 to 0.77, *P* < 0.0001, I2 = 50.8%, Figure 3). Leave-one-out sensitivity analysis did not materially change the direction of effect. However, moderate heterogeneity remained, and postoperative atelectasis was not assessed uniformly across studies with respect to timing, diagnostic thresholds, or lung ultrasound scoring methods.

Subgroup analyses (Supplementary Material 3) suggested a significant effect in abdominal surgery (RR, 0.62, 95% CI, 0.52 to 0.73, I2 = 0%), but not in thoracic surgery (RR, 0.69, 95% CI, 0.45 to 1.08, I2 = 59.7%). Ultrasound-guided recruitment also favored both comparator categories (non-ultrasound-guided recruitment maneuvers: RR, 0.74, 95% CI, 0.61 to 0.90, I2 = 36.3%; standard lung-protective ventilation: RR, 0.51, 95% CI, 0.39 to 0.67, I2 = 0%), but the non-ultrasound-guided recruitment maneuvers subgroup was clinically more heterogeneous because conventional recruitment protocols differed across trials.

### Secondary outcomes: PPCs and length of hospital stay

**Figure 4:**
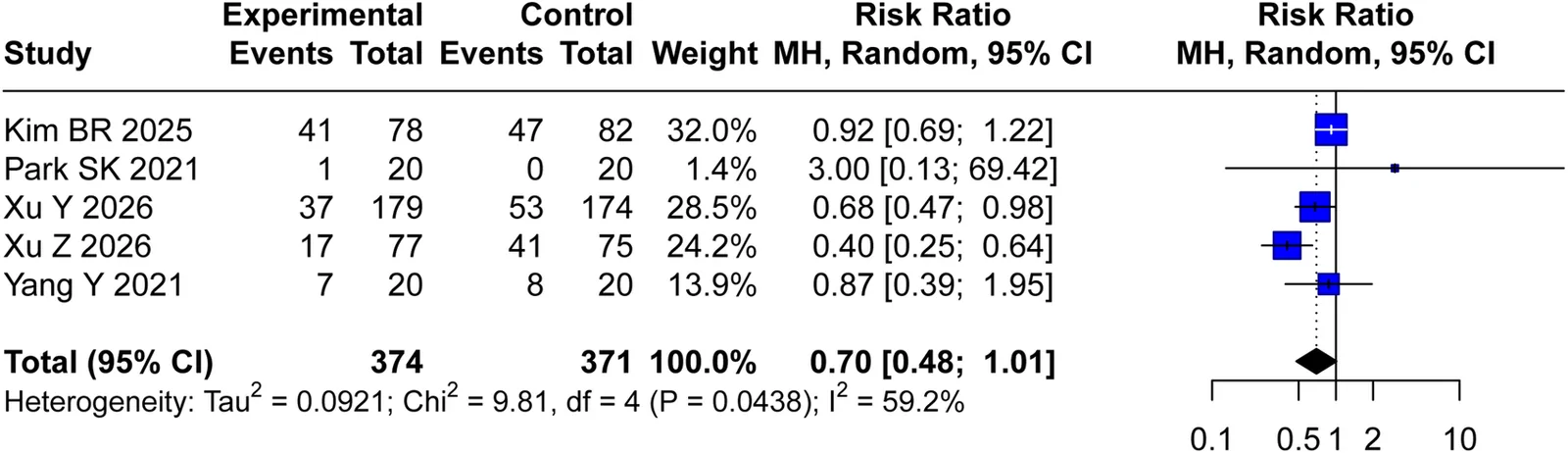
Forest plot comparing the effect of ultrasound-guided lung recruitment maneuvers on incidence of postoperative pulmonary complications.

**Figure 5:**
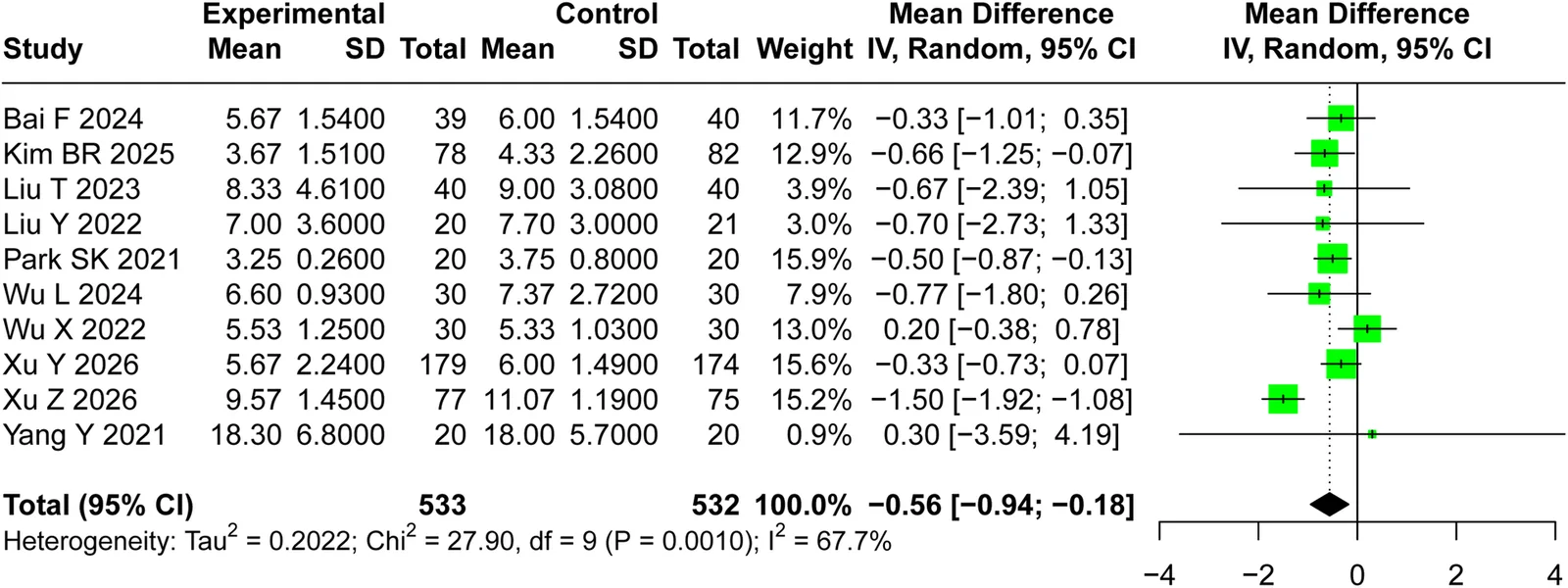
Forest plot comparing the effect of ultrasound-guided lung recruitment maneuvers on length of hospital stay.

For PPCs, the pooled result did not reach statistical significance (RR, 0.70, 95% CI, 0.48 to 1.01, *P* = 0.0571, I2 = 59.2%, Figure 4). Length of hospital stay was statistically shorter with ultrasound-guided recruitment (MD, −0.56 days, 95% CI, −0.94 to −0.18, *P* = 0.0039, I2 = 67.7%, Figure 5), but the absolute reduction was modest.

### Sensitivity analyses

Sensitivity analyses (Supplementary Material 3) suggested that the PPC estimate was influenced by individual studies, including Kim BR et al. (11), whereas the length-of-stay estimate remained directionally consistent. Because PPC definitions and discharge practices varied across trials, both secondary outcomes require cautious interpretation. Subgroup analyses (Supplementary Material 3) suggested ultrasound-guided lung recruitment maneuvers reduced the incidence of PPCs in abdominal surgery (RR, 0.61, 95% CI, 0.40 to 0.92, I2 = 40.5%), but not in thoracic surgery. The effect of ultrasound-guided lung recruitment maneuvers on length of hospital stay reduction remained consistent across various surgical procedures and control strategies, with no significant differences observed between subgroups.

### GRADE assessment

Using GRADE, the certainty of evidence was rated as low for postoperative atelectasis and length of hospital stay and very low for PPCs (Supplementary Material 4). Downgrading was mainly driven by residual heterogeneity, suspected small-study effects or publication bias, imprecision for PPCs, indirectness related to geographic and surgical concentration, and variability in ultrasound and ventilation protocols.

## Discussion

### Principal findings

This meta-analysis of 10 RCTs suggests that ultrasound-guided lung recruitment maneuvers may reduce postoperative atelectasis, particularly in abdominal surgery. However, the cumulative evidence base is small, several trials enrolled few participants, and important clinical and methodological heterogeneity remains. The pooled estimates should therefore be interpreted as suggestive rather than definitive.

The secondary outcomes are less certain. PPCs were not significantly reduced in the primary analysis, and the statistically significant reduction in hospital stay was modest in absolute terms. These findings may be relevant for perioperative pathways if confirmed in larger trials, but they should not be overinterpreted as evidence of major clinical or economic benefit.

### Comparison with existing literature

Our findings extend and update the prior systematic review by Liao et al. (14), which similarly demonstrated that ultrasound-guided lung recruitment maneuvers improved lung aeration scores. The present review incorporates more recent trials and explores outcomes by surgical type and comparator strategy. These subgroup findings are exploratory and should not be interpreted as demonstrating effect modification because the number of studies was limited and formal meta-regression was not feasible.

Residual heterogeneity is central to the interpretation of this review. Included trials differed in recruitment maneuver protocols, ultrasound guidance techniques, pressure targets, timing of recruitment, maintenance ventilation and post-recruitment PEEP strategies, surgical populations, and outcome definitions. These differences may modify the treatment effect and limit the comparability of pooled estimates. For example, a trial using individualized PEEP after recruitment may not estimate the same intervention effect as a trial that returns to fixed PEEP after a single ultrasound-guided maneuver.

The apparent difference between abdominal and thoracic surgery should also be interpreted cautiously. Abdominal surgery may produce more predictable dependent atelectasis related to pneumoperitoneum and supine positioning, whereas thoracic surgery and one-lung ventilation involve deliberate lung collapse, pleural disruption, surgical trauma, and different ventilation constraints (30). The thoracic subgroup included fewer studies and showed residual heterogeneity; specialized protocols may be required before conclusions can be drawn for this population.

Operator dependency is another important limitation. Ultrasound-guided recruitment requires image acquisition, interpretation of aeration patterns, and translation of findings into ventilator adjustments. Postoperative atelectasis diagnosis by lung ultrasound is similarly dependent on scanning technique, scoring system, and assessor training. Because the included studies did not consistently report operator expertise or interobserver reliability, we could not evaluate whether ultrasound competency influenced the pooled effects. This may affect both internal validity and generalizability to centers with less point-of-care ultrasound experience.

The PPC and publication-bias findings require particular caution. PPC definitions varied across trials, and the primary PPC analysis was borderline and statistically heterogeneous. Although trim-and-fill analysis changed the PPC estimate, this method is exploratory and relies on assumptions that may not hold when heterogeneity is present. It should therefore be viewed as a sensitivity analysis indicating uncertainty, not as confirmation of a true PPC benefit. The reduction in hospital length of stay should also be interpreted conservatively. The pooled MD was approximately half a day, and its clinical relevance depends on baseline risk, discharge criteria, health-system context, and perioperative care pathways. This outcome may be vulnerable to non-respiratory influences that were not consistently controlled across trials.

Two reports by Cylwik and Buda, although excluded from the quantitative synthesis, provide relevant contextual evidence from a Polish surgical population. Their single-group monitoring study showed that lung ultrasound could guide individualized recruitment and PEEP selection (31), while their subsequent two-group report described fewer ICU admissions, less prolonged postoperative mechanical ventilation, and fewer respiratory infections after ultrasound-guided recruitment with individualized PEEP (32). These observations support the clinical plausibility of the intervention and extend the geographic context beyond East Asia; however, the single-arm design of the earlier study, mixed elective and emergency population and insufficiently reported allocation methods in the later study, and absence of outcomes matching our prespecified definitions precluded inclusion in the meta-analysis.

The durability of recruitment is also likely influenced by the maintenance ventilation strategy after the maneuver. Recruitment maneuvers can reopen dependent or collapsed lung regions, but re-collapse may occur when the post-recruitment PEEP is insufficient to maintain end-expiratory lung volume. Among the included studies, only Xu et al. (13) clearly incorporated individualized PEEP titration to minimize driving pressure, while Liu et al. (23) evaluated ultrasound-guided recruitment in combination with PEEP and most remaining studies used fixed PEEP levels or returned to prior ventilator settings. This variability limits our ability to isolate the effect of ultrasound guidance from the effect of subsequent PEEP optimization. Future trials should report opening pressure, closing pressure when available, post-recruitment PEEP selection, and adherence to maintenance ventilation protocols.

### Clinical implications

Clinically, the current evidence supports considering ultrasound-guided recruitment as an adjunct in selected abdominal surgery patients when trained operators and appropriate workflow are available. The evidence does not support broad routine implementation across all surgical populations. Practical adoption should account for training requirements, equipment availability, intraoperative workflow, and predefined protocols linking ultrasound findings to recruitment pressure and post-recruitment PEEP.

Future trials should use standardized lung ultrasound scoring and atelectasis definitions, report operator training and interobserver reliability, specify recruitment timing and pressure algorithms, and define post-recruitment PEEP strategies. Multicenter trials outside East Asia and in higher-risk populations are needed to improve precision and external validity.

### Limitations

Several limitations should be emphasized. First, the evidence base included only 10 RCTs and 1,076 patients, with some small single-center studies; this limits precision, robustness, and the ability to explore effect modifiers. Second, residual statistical and clinical heterogeneity was substantial despite random-effects modeling. Third, the prespecified subgroup analyses and meta-regression according to operator expertise, timing of postoperative atelectasis assessment, diagnostic criteria, and lung ultrasound scoring systems could not be performed because reporting was inconsistent, categories were sparse, and only ten trials were available. This limited further exploration of important sources of clinical heterogeneity and precluded assessment of whether these clinically relevant variables modified the observed treatment effects. Operator training, formal competency assessment, and interobserver reliability were also incompletely reported, which limits interpretation and generalizability and may have introduced performance or measurement bias. Fourth, postoperative atelectasis was assessed using different time points, diagnostic criteria, and ultrasound scoring systems, further limiting comparability across studies. Fifth, suspected small-study effects and publication bias were present, and trim-and-fill results were exploratory. Sixth, all trials were conducted in China or Korea, limiting generalizability to other healthcare systems. Two potentially relevant reports were identified only during peer review, indicating limited sensitivity of the original search. Both were excluded after full-text reassessment for predefined design, population, or outcome reasons; nevertheless, the possibility that other relevant published evidence was missed cannot be excluded. Finally, most trials enrolled elective low- to moderate-risk patients and had short follow-up, so applicability to high-risk patients, ICU-level care, and long-term recovery remains uncertain.

## Conclusion

In conclusion, ultrasound-guided lung recruitment maneuvers may reduce postoperative atelectasis in adults undergoing elective surgery, with the clearest signal in abdominal surgery. However, the evidence remains limited by a small number of trials, heterogeneous protocols and outcome definitions, operator dependency, possible small-study effects, and low or very low certainty for major outcomes. The modest reduction in hospital stay should not be overstated, and current data are insufficient to support broad routine implementation. Larger, standardized, multicenter RCTs with transparent reporting of ultrasound expertise, recruitment protocols, post-recruitment PEEP, and patient-centered outcomes are needed.
