# Supplementary material for: The effect of ultrasound-guided lung recruitment maneuvers on postoperative atelectasis: a systematic review and meta-analysis of randomized controlled trials
Source: Front Cardiovasc Med. 2026 Aug 12;13:1890372. doi: 10.3389/fcvm.2026.1890372 (PMC13507955; doi:10.3389/fcvm.2026.1890372)
Supplement: Supplementary file 1 [file Datasheet1.docx]

Supplementary Material 1: PRISMA checklist

**Supplementary Material 2: Searching strategies**

**Pubmed 109**

#1 recruitment [Title/Abstract] OR pulmonary reexpansion [Title/Abstract] OR lung reexpansion [Title/Abstract]

#2 Ultrasonography [MeSH Terms] OR Ultrasonics [MeSH Terms] OR Ultrasonography [Title/Abstract] OR ultrasound [Title/Abstract] OR ultrasonographic [Title/Abstract] OR Ultrasound-guided [Title/Abstract]

#3 Postoperative Complications [MeSH Terms] OR Postoperative Period [MeSH Terms] OR Postoperative [Title/Abstract] OR Post-operative [Title/Abstract] OR surgery [Title/Abstract]

#4 randomized controlled trial [MeSH Terms] OR randomized [Title/Abstract] OR randomised [Title/Abstract] OR random* [Title/Abstract]

#1 AND #2 AND #3

**Embase 334**

#1 ‘recruitment’:ti,ab,kw OR ‘reexpansion’:ti,ab,kw

#2 ' Ultrasonography ':ti,ab,kw OR ‘Ultrasonics’:ti,ab,kw OR ‘ultrasound’:ti,ab,kw OR ‘ultrasonographic’:ti,ab,kw

#3 ' Postoperative ':ti,ab,kw OR ‘Post-operative’:ti,ab,kw OR ‘surgery’:ti,ab,kw

#4 'randomized controlled trial'/de OR 'randomized controlled trial'/exp

#1 AND #2 AND #3 AND #4

**Scopus 142**

#1 TITLE-ABS-KEY (recruitment) OR TITLE-ABS-KEY (pulmonary reexpansion) OR TITLE-ABS-KEY (lung reexpansion)

#2 TITLE-ABS-KEY (Ultrasonography) OR TITLE-ABS-KEY (Ultrasonics) OR TITLE-ABS-KEY (ultrasound) OR TITLE-ABS-KEY (ultrasonographic)

#3 TITLE-ABS-KEY (Postoperative) OR TITLE-ABS-KEY (Post-operative) OR TITLE-ABS-KEY (surgery)

#4 TITLE-ABS-KEY (randomized) OR TITLE-ABS-KEY (random) OR TITLE-ABS-KEY (randomised)

#1 AND #2 AND #3 AND #4

**Cochrane Library 376**

#1 (recruitment):ti,ab,kw OR (reexpansion):ti,ab,kw

#2 (Ultrasonography):ti,ab,kw OR (Ultrasonics):ti,ab,kw OR (ultrasound):ti,ab,kw OR (ultrasonographic):ti,ab,kw

#3 (Postoperative):ti,ab,kw OR (Post-operative):ti,ab,kw OR (surgery):ti,ab,kw

#4 (randomized):ti,ab,kw OR (randomised):ti,ab,kw OR (random):ti,ab,kw

#1 AND #2 AND #3 AND #4

**Supplementary Material 3:** Publication bias assessment by funnel plot and Egger’s test, forest plots for sensitivity analyses and subgroup analysis

Figure 1: Publication bias assessment by funnel plot and Egger’s test (A) incidence of postoperative atelectasis, Egger’s test P=0.0074; (B) incidence of postoperative pulmonary complications; (C) length of hospital stay, Egger’s test P=0.7673

Figure 2: Forest plot for incidence of postoperative atelectasis after trim and fill method

Figure 3: Forest plots for incidence of postoperative pulmonary complications after trim and fill method

Figure 4: Forest plots of sensitivity analyses for (A) incidence of postoperative atelectasis; (B) incidence of postoperative pulmonary complications; (C) length of hospital stay

Figure 5: Subgroup analysis for incidence of postoperative atelectasis stratified by (A) surgical type, thoracic surgery versus abdominal surgery; (B) control group intervention, non-ultrasound-guided lung recruitment maneuvers versus standard protective mechanical ventilation

Figure 6: Subgroup analysis for incidence of postoperative pulmonary complications stratified by (A) surgical type, thoracic surgery versus abdominal surgery; (B) control group intervention, non-ultrasound-guided lung recruitment maneuvers versus standard protective mechanical ventilation

Figure 7: Subgroup analysis for length of hospital stay stratified by (A) surgical type, thoracic surgery versus abdominal surgery; (B) control group intervention, non-ultrasound-guided lung recruitment maneuvers versus standard protective mechanical ventilation

**Supplementary Material 4:** GRADE certainty-of-evidence assessment

| Outcome | Studies / participants | Effect estimate | Certainty | Main reasons for rating |
| --- | --- | --- | --- | --- |
| Postoperative atelectasis | 10 RCTs / 1,076 patients | RR 0.64 (95% CI 0.52 to 0.77) | Low | Downgraded for residual clinical/statistical heterogeneity and suspected small-study effects/publication bias. Additional concerns included variation in ultrasound protocols, recruitment timing, and outcome assessment. |
| PPCs | 5 RCTs / 793 patients | RR 0.70 (95% CI 0.48 to 1.01) | Very low | Downgraded for inconsistency, imprecision because the confidence interval included no effect, suspected small-study effects/publication bias, and heterogeneous PPC definitions. |
| Length of hospital stay | 10 RCTs / 1,060 patients | MD -0.56 days (95% CI -0.94 to -0.18) | Low | Downgraded for substantial heterogeneity and indirectness related to differences in surgical populations, discharge practices, perioperative pathways, and geographic concentration of included trials. |

CI: confidence interval; MD: mean difference; PPCs: postoperative pulmonary complications; RCT: randomized controlled trial; RR: risk ratio.
